# Assessing change in patient-reported quality of life after elective surgery: protocol for an observational comparison study

**DOI:** 10.12688/f1000research.8758.1

**Published:** 2016-05-24

**Authors:** Vanessa L. Kronzer, Michelle R. Jerry, Michael S. Avidan

**Affiliations:** 1 Department of Anesthesia, Washington University School of Medicine, Saint Louis, MO, 63110, USA; 2 Department of Biostatistics, University of Michigan, Canton, MI, 48188, USA

**Keywords:** quality of life, surgical procedures, outcome assessment, postoperative period

## Abstract

Despite their widespread use, the two main methods of assessing quality of life after surgery have never been directly compared. To support patient decision-making and study design, we aim to compare these two methods. The first of these methods is to assess quality of life before surgery and again after surgery using the same validated scale. The second is simply to ask patients whether or not they think their post-operative quality of life is better, worse, or the same. Our primary objective is to assess agreement between the two measures. Secondary objectives are to calculate the minimum clinically important difference (MCID) and to describe the variation across surgical specialties. To accomplish these aims, we will administer surveys to patients undergoing elective surgery, both before surgery and again 30 days after surgery. This protocol follows detailed guidelines for observational study protocols.

## Background

The following protocol follows published guidelines for observational study protocols ^1^. The research question for this study is how validated measures compare to self-reported measures of quality of life in patients undergoing elective surgery. To answer this question, we performed a literature search in PubMed.

Research studying patient-reported quality of life is burgeoning, including quality of life related to surgery ^2, 3^. Two main methods are used to estimate a procedure’s impact on quality of life. The first is to compare patient scores before and after surgery using a validated quality of life scale ^4, 5^. The second method is to ask patients about their change in quality of life after surgery occurs ^6– 8^. This type of self-reported “global measure” is growing in popularity ^9^. However, patients’ perceived change in quality of life may be inaccurate due to cognitive biases such as choice-supportive bias ^10, 11^, or theory-driven recall bias ^12, 13^. Uncovering potential bias in measures of quality of life is important since patients and clinicians base their surgical decision-making on these measures.

In addition, the minimum clinically important difference (MCID) in quality of life has been established in the literature for general populations ^14, 15^ and for neurosurgical populations ^16, 17^, but has not been studied in general surgical populations. Establishing a MCID for quality of life is crucial, both for patients deciding whether or not to receive surgery and for clinicians evaluating the effectiveness of surgery.

With its large sample size and general population of elective surgery patients, this study is uniquely poised to compare the two methods of patient-reported change in quality of life and to determine the difference in quality of life score that surgical patients can detect. Determining the most accurate way to ascertain patient-reported quality of life can support elective surgery decisions and future studies of quality of life.

## Specific aims

Aim 1

The primary aim of this study is to compare self-reported change in quality of life (better/same/worse) to the change in a validated (VR-12) quality of life score (both physical and mental component scores), 30 days after elective surgery.

We hypothesize that the median physical and mental quality of life scores will be significantly higher in patients reporting “better” quality of life compared to those reporting “same,” and significantly higher in those reporting “same” quality of life to those reporting “worse.” We also hypothesize that the overall agreement will be “substantial” (kappa=0.61 to 0.80), with the majority of error occurring in patients whose validated measure showed a decline in quality of life, but who reported “same” or “better” quality of life. For that reason, we expect the percent agreement between the self-reported and validated scales to be lowest in the group reporting “better” quality of life after surgery.

Aim 2

A secondary aim is to compare the change in physical and mental quality of life scores that patients were able to perceive to the MCID established in literature.

We hypothesize that the difference in quality of life that our surgical patients can detect will be similar to the difference reported in the literature (ie a 5-point change).

Aim 3

Another secondary aim is to describe the change in physical and mental quality of life for both methods across surgical specialties.

We hypothesize that the change in quality of life will be greater for specialties correcting limited problems such as orthopedic and plastic surgery, while the change will be lower for specialties with more complex problems such as neurosurgery and cardiothoracic surgery.

## Study design

This prospective, observational cohort study is a sub-study of the Systematic Assessment and Targeted Improvement of Services Following Yearlong Surgical Outcomes Surveys (SATISFY-SOS) study. SATISFY-SOS is an ongoing registry that has been enrolling patients at Barnes Jewish Hospital since July, 2012 ^18^. All enrolled patients complete a survey of baseline health during their visit to the preoperative assessment clinic and then complete a follow-up survey approximately 30 days after surgery (see Supplementary Material for these two surveys). The intervention for this study is to compare self-reported quality of life (“How would you rate your quality of life now? (better/same/worse)”) to the quantitative change in their VR-12 quality of life scores between the baseline survey and 30-day follow-up survey. All patients answer both questions and therefore serve as their own controls.

## Study group

The target population is all patients undergoing elective surgery at Barnes Jewish Hospital who attended the center for preoperative assessment and planning between January 15, 2014 and October 7, 2015. Inclusion criteria include age 18 or older, ability to read the English consent form (see Supplementary Material for the consent form), ability to consent, and plans to undergo elective surgery. Over 70% of patients undergoing elective surgery are assessed by the center for preoperative assessment and planning (CPAP) clinic before surgery. Reasons for no assessment include urgent surgery, geographical limitations, or surgeon preference. Approximately 65% of all eligible patients consent to participate in SATISFY-SOS. Reasons for non-participation include patient refusal ( ^~^70% of cases), lack of nurse time or training ( ^~^20%), or lack of English literacy ( ^~^10%). A study comparing participants to non-participants showed no major differences in characteristics ^19^. Approximately 92% of consented patients complete the baseline survey, and 60% of those respond to the 30-day follow-up survey.

A total of 9,097 “complete” records (with both baseline and 30-day surveys) are available in the proposed time window. For the purposes of this sub-study, only the first complete record for each will be included in the final dataset (approximately 94% of the available records). This practice ensures that each record is statistically independent from all the other records. In addition, records with surgery to 30-day response dates of less than 20 days or more than 120 days will be excluded.

## Recruitment

Nurses at the CPAP clinic assess patient eligibility, recruit patients to participate, and obtain written consent. No payment is provided. While most patients decide whether or not to participate at this time, a patient can decide to participate any time between his or her CPAP visit and his or her surgery day. For patients who need special assistance, such as those who are blind or cannot physically sign a form, a witness can be obtained. However, in practice this rarely occurs. No arrangements are made for non-English speakers, mentally ill, children, or those suffering from dementia since those are excluded groups. If patients agree to participate, the CPAP nurse asks them to complete the baseline survey at the time of consent. Approximately 30 days following surgery, they receive a similar follow-up survey. Both surveys were designed to take 10 to 15 minutes to complete. The SATISFY-SOS research team holds monthly update meetings with all CPAP nurses to inform them about study progress and to encourage optimal recruitment.

## Data

All preoperative and postoperative quality of life data comes from the SATISFY-SOS surveys, which are administered to patients at the preoperative assessment visit and then approximately 30 days after surgery. To maximize the follow-up survey response rate, patients are emailed the survey (once), mailed hard copy surveys (two times), and phoned (up to five times). The twelve items comprising the Veterans RAND 12 (VR-12) are items 24 through 35 on the survey, while the self-reported global quality of life question is item 1. The VR-12 is made up of two components: a physical component score (PCS), and a mental component score (MCS). Both scores are continuous on a scale from 0 to 100 (where higher is greater quality of life), and they are calibrated so that a score of 50 represents the US population mean ^20, 21^. The 30-day follow-up questionnaires also ask patients to self-report their change in quality of life. The question asks, “How would you rate your quality of life **now**?” with answer choices including “Better than before your procedure,” “The same as before your procedure,” and “Worse than before your procedure.” Surgical specialty for the procedure is obtained from the electronic medical record. Using queries in MetaVision (iMDsoft, Needham, MA), the informaticist will provide the requested survey and medical record data to the investigators. He performs rigorous data validation on each queried variable.

SATISFY-SOS databases are hosted on a firewall-secured network server managed by the Department of Anesthesiology. The server is securely housed behind two locked doors within the departmental office suite and maintained and managed by the departmental IT team. Only the project Informaticist, Data Manager, and Director(s) have full access to these databases, which are also password-protected and encrypted for additional protection. Hardcopies of the baseline surveys are collected daily from the CPAP clinic and securely stored behind two locked doors within the Department of Anesthesiology. Baseline completed paper surveys are scanned into a digital image format (compressed TIFF). The digital image files are indexed and stored on a research file server that is attached to a private network with no public access. Survey email, mail and call lists are generated at Washington University in a similar manner to mailing lists for billing services. For each patient and date of service, a unique ID is generated and never duplicated. This unique ID is a nonsensical only meaningful to the research team.

Baseline surveys are processed by Solutions Data Systems. The digital image files are transmitted to Solutions Data Systems via secure file transfer protocol. When data entry has been confirmed, Solutions Data Systems deletes the digital image file from their servers. Press Ganey *,* a vendor specializing in patient survey distribution and collection, disseminates, collects, and processes 30-day and 1-year surveys. Paper surveys processed through automated scanning are all manually checked, and a manager listens to 10% of telephone surveys. All telephone surveys are recorded and available for future quality checks for performance improvement. Press Ganey stores the survey hardcopies for 90 days while the study team conducts spot-check quality assessments of the scanned data. The company then shreds the paper copies. Similarly, Press Ganey will hold copies of the electronic files and electronic recordings for 90 days, after which the electronic files are removed permanently from their system (and then only maintained by Washington University). During this 90-day period, the study team conducts additional quality assessments of the converted data.

## Statistical considerations

We base sample size considerations for this study on the primary outcome. The first component of the primary endpoint is comparing the change in VR-12 quality of life scores among the three self-reported change groups (better/same/worse). Using a minimum important difference of five points, two tails, alpha of 0.05, and 80% power, the required sample size is 77 patients per group, or 231 total patients among the three groups. The second component of the primary outcome is the agreement between the two quality of life measures, as reflected by the kappa statistic. Kappa does not have sample size requirements beyond lack of sparse cells. The third component of the primary outcome is comparing the percentage agreement across the three different self-reported change groups. Since no studies have performed this type of comparison previously, we pre-specified a 10% change as the minimum important change. Estimating 80% agreement, and using two tails, alpha=0.05, and 80% power, the required total sample size is 311 per group, or 933 total patients. Therefore, this study has adequate power for all of these endpoints.

The following statistical analyses will be performed, using alpha=0.05 and 95 percent confidence intervals, where appropriate. All analyses will be performed twice, once for VR-12 physical quality of life, and again for the VR-12 mental quality of life.

Aim 1:

- Compare change in VR-12 QOL scores for those answering better/same/worse (Kruskal-Wallis). If significant, will use Wilcoxon Rank-Sum tests to compare each of the three groups, using a Bonferroni correction of alpha=0.017.
- Calculate overall agreement between the self-reported and validated quality of life measures (using weighted kappa, which penalizes disagreements in proportion to their seriousness, see Table 1) ^22^.
- Calculate the overall percent of patients whose self-reported and validated quality of life scores matched (descriptive), including stratification by self-reported global change better/same/worse (compared using chi-square). For the purpose of this study, “matching” consists of:
  ◦ Change in VR-12 > 0 for those responding their quality of life was “better”
  ◦ Change in VR-12 between +5 and -5 for those responding their quality of life was the “same”
  ◦ Change in VR-12 < 0 for those responding their quality of life was “worse”

**Table 1.** Weights for weighted kappa calculation.

	Change in VR-12 Score between baseline and 30-days				
Self-Reported Quality of Life	<-5	-5 to 0	0	0 to +5	>+5
Better than before procedure	0	0.33	0.66	1	1
Same compared to before	0.33	1	1	1	0.33
Worse than before procedure	1	1	0.66	0.33	0

Aim 2:

- Calculate the quality of life where an equal proportion of patients reported better and same quality of life (MCID for improvement), or same and worse quality of life (MCID for deterioration). This is an anchor-based approach ^23^.

Aim 3:

- Describe the change in VR-12 score and percent of patients reporting better, same, and worse quality of life for each of the following surgical specialties: neurosurgery, orthopedic, plastic, ophthalmologic, general, cardiac, gynecologic, otolaryngology, gastrointestinal/hepatobiliary, urologic, and “other.”

Only those answering both the self-reported quality of life question and at least ten out of twelve VR-12 questions at baseline and 30 days will be included. We will describe the characteristics of those with and without missing data. Multiple imputation will be used to fill in missing measurements for those missing two or fewer items on the VR-12 questionnaire. Analysis will be performed by VLK and MRJ after this protocol is submitted online.

## Limitations

Although the “validated” VR-12 measure is based on a standardized scale that has been rigorously tested and studied, it is also self-reported and probably also contains bias. Because the VR-12 produces physical and mental component scores (PCS and MCS), but the self-reported question asks about overall quality of life, the observed association between the self-reported quality of life and the individual component scores might be artificially lowered. Also, the order of the questions on the surveys might influence patients’ responses by priming them. Since the self-reported question occurs first, it may alter responses on the VR-12 items, which occur later in the same survey. Another limitation is that postoperative quality of life is only measured at 30 days. It is possible that the relationship between the two measures is different at different time points. In addition, the 30-day follow-up survey is often completed near 30 days, but the time of completion ranges from 20 to 120 days after surgery. Nevertheless, the time point for completing the two quality of life scales is the same in each individual patient, so the main results of this study should not be affected.

This study includes just one academic medical center, and its patient population and rules for preoperative assessment clinic attendance may differ from other hospitals. In addition, only 65% of eligible preoperative assessment clinic attendees enrolled in the study, which may introduce bias. However, our analyses indicate that participants do not differ in important ways from non-participants ^19^. Furthermore, even if the sample was maximally biased, enrolling 65% of the actual target population means the results contain at least 80% accuracy ^24^. Another factor that biases the sample is nonresponse to the follow-up surveys. Though we mitigate nonresponse through an extensive follow-up protocol, only 60% of patients respond to the 30-day survey. Fortunately, our previous work shows that the characteristics of responders do not differ significantly from the characteristics of non-responders (unpublished manuscript). Finally, anchor-based approaches for calculating MCID can be influenced by recall bias and have been shown to more strongly reflect patients’ current health status than the true change over time ^23.^

## Compliance

Since the exposure for this study is patient-reported quality of life, no procedures for monitoring exposure compliance are necessary. Subjects are withdrawn from SATISFY-SOS if requested. The informaticist and Press Ganey are notified to ensure that the patient is no longer approached for data collection. As described in the consent form, data already collected may continue to be used.

## Ethical considerations

This study is approved by the Institutional Review Board (Washington University Human Research Protection Office, IRB ID# 201505035). As a sub-study of SATISFY-SOS, it has a waiver of informed consent. Written, informed consent is obtained from all participants for SATISFY-SOS (IRB ID# 201203088). Since this study is survey-based, it involves no more than minimal risk to patients. As described above, no special allowances are made for non-English speakers, children, or mentally ill. Participants may withdraw from the study at any time.

## Finance and insurance

Finance details, insurance details, and cover for negligent and non-negligent harm are not relevant for this study since it involves no more than minimal risk to patients. Patients receive no compensation for participation.

## Reporting and dissemination

Results of this study will be presented at national meetings and published in a scientific journal. Participants will be individually notified of results only if discoveries are made that directly impact their health. The data and code for this project will be available upon email request.

## Supplementary Material

**Baseline survey.**

Questionnaire provided to patients during their preoperative assessment visit

.

**30-day survey.**

Follow-up questionnaire sent to patients approximately 30 days after surgery

.

**Consent form.**

Consent form for SATISFY-SOS.
