# Supplementary material for: Assessing change in patient-reported quality of life after elective surgery: protocol for an observational comparison study
Source: F1000Res. 2016 May 24;5:976. [Version 1] doi: 10.12688/f1000research.8758.1 (PMC5017283; doi:10.12688/f1000research.8758.1)
Supplement: Supplementary file 1 [file f1000research-5-9425-s0000.tgz › 24427644-e084-470f-a147-f32c5e92e182.rtf]

Please provide one answer for each question. Please circle your answer. If you are unsure how to answer a question, please choose the answer that fits best. 
1.	Why are you having this upcoming surgery? (Circle all that apply)
•	Treat or cure a medical condition
•	Decrease pain 
•	Treat my symptoms
•	Improve my ability to perform daily life activities at home
•	Improve my abilities to perform daily life activities away from home
•	Improve my quality of life
•	Prolong my life
•	Doctor said I needed the surgery

2.	Do you have pain now? (Check all that apply)
•	Yes, but NOT RELATED to my need for surgery
•	Yes, and RELATED to my need for surgery
•	No
•	Don't know

3.	What is your expectation about pain a month after your surgery?
•	I have pain now and expect to have less pain after surgery
•	I have pain now and expect it to stay the same after surgery
•	I have pain now but I expect to have more pain after surgery
•	I have no pain now but I expect to have pain after surgery
•	I have no pain now and I do not expect to have pain after surgery
•	Don't know

4.	In your opinion, how long would it take you to return to you normal life activities after you surgery:
•	Less than 1 week
•	1-4 weeks
•	1-3 months
•	3-6 months
•	More than 6 months
•	
This section is about your general health. These questions do not necessarily relate to your upcoming procedure.
5.	In the past six months, how many times have you had a fall, including a slip or trip in which you lost your balance and landed on the floor or ground or lower level?
•	Zero (0) (Please skip to question #6)
•	One time (1)
•	Two times (2)
•	Three or more (>2)

6.	Did your fall result in any of the following? (Circle all that apply)
•	No injury
•	Bruising, sprains or cuts
•	Reduced mobility
•	A fear of falling
•	Severe pain
•	Injury causing you to seek medical treatment
•	Broken bone
•	Head injury
•	A change from independent living to assisted living

7.	 In general, would you say your health is:  
•	Excellent
•	Very good
•	Good
•	Fair
•	Poor

8.	 Compared to one year ago, how would you rate your physical health in general now?
•	Much better
•	Slightly better
•	About the same
•	Slightly worse
•	Much worse     

9.	 Compared to one year ago, how would you rate your emotional health now? (Such as feeling anxious, depressed or irritable) 
•	Much better
•	Slightly better
•	About the same
•	Slightly worse
•	Much worse

10.	Does your health now limit you in moderate activities, such as moving a table, pushing a vacuum cleaner, bowling, or playing golf? If so, how much?	
•	Yes, limited a lot
•	Yes, limited a little
•	No, not limited at all

11.	Does your health now limit you in climbing several flights of stairs? If so, how much?	
•	Yes, limited a lot
•	Yes, limited a little
•	No, not limited at all

12.	As a result of your physical health, during the past 4 weeks, have you accomplished less than you would like with your work or other regular daily activities? 
•	No, none of the time
•	Yes, a little of the time
•	Yes, some of the time
•	Yes, most of the time
•	Yes, all of the time  

13.	As a result of your physical health, during the past 4 weeks, were you limited in the kind of work or other activities you can perform? 
•	No, none of the time
•	Yes, a little of the time
•	Yes, some of the time
•	Yes, most of the time
•	Yes, all of the time

14.	As a result of any emotional problems (such as feeling depressed or anxious), during the past 4 weeks, have you accomplished less than you would like with your work or other regular daily activities? 
•	No, none of the time
•	Yes, a little of the time
•	Yes, some of the time
•	Yes, most of the time
•	Yes, all of the time

15.	 As a result of emotional problems (feeling depressed or anxious), during the past 4 weeks, have you not done work or other activities as carefully as usual? 	
•	No, none of the time
•	Yes, a little of the time
•	Yes, some of the time
•	Yes, most of the time
•	Yes, all of the time

16.	During the past 4 weeks, how much did pain interfere with your normal work (including both work outside the home and housework)?  
•	Not at all
•	A little bit
•	Moderately
•	Quite a bit
•	Extremely

17.	How much of the time during the past 4 weeks have you felt calm and peaceful? 	
•	All of the time
•	Most of the time
•	A good bit of the time
•	Some of the time
•	A little bit of the time
•	None of the time

18.	How much of the time during the past 4 weeks did you have a lot of energy?
•	All of the time
•	Most of the time
•	A good bit of the time
•	Some of the time
•	A little bit of the time
•	None of the time

19.	How much of the time during the past 4 weeks have you felt downhearted and blue?	
•	All of the time
•	Most of the time
•	A good bit of the time
•	Some of the time
•	A little bit of the time
•	None of the time

20.	How much of the time during the past 4 weeks has your physical health or emotional problems interfered with your social activities (like visiting with friends, relatives, etc.)?
•	All of the time
•	Most of the time
•	Some of the time
•	A little bit of the time
•	None of the time

21.	 If your ability to perform work is 10 when you are at your best and 0 when you are unable to work, circle the number that represents your ability to work this past week. 
0    1    2    3    4    5    6    7    8    9    10  

22.	What is your work status? 
•	Employed 
•	Volunteer work 
•	Unemployed
•	Student
•	Homemaker
•	Retired (END OF SURVEY)
•	Disabled (END OF SURVEY)
23.	Does your health limit you in your current job (work or studies or housework)? 
•	I am not limited by my health
•	I am able to do my job with difficulty
•	I sometimes have to work slowly
•	I often have to work slowly
•	I am only able to do my job part time
•	I am entirely unable to do my job 

24.	Do you believe that, from the standpoint of your health, you will be able to do your current job in 1 year?
•	Yes
•	No
•	Not sure
	
