# Supplementary material for: Assessing change in patient-reported quality of life after elective surgery: protocol for an observational comparison study
Source: F1000Res. 2016 May 24;5:976. [Version 1] doi: 10.12688/f1000research.8758.1 (PMC5017283; doi:10.12688/f1000research.8758.1)
Supplement: Supplementary file 2 [file f1000research-5-9425-s0001.tgz › f6a77036-4660-415a-be15-24f049fe3611.rtf]

											


1.	How would you rate your quality of life now?
·	Better than before your procedure	 
·	The same as before your procedure		
·	Worse than before your procedure

2.	Since your surgery, have you been able to return to work? 
·	Yes  	 
·	No	
·	Does not apply

3.	While still in the hospital after your recent surgery, did you have PROBLEMS WITH YOUR HEART:
(Fill in all that apply)
·	Heart attack?
·	Your heart stopped beating (cardiac arrest)?
·	Heart failure (congestive heart failure)?
·	Abnormal heart rhythm such as atrial fibrillation?
·	Severe pain coming from your heart (angina)?
·	None

4.	While still in the hospital after your recent surgery, did you have PROBLEMS WITH BLOOD CLOTS:  (Fill in all that apply)
·	Blood clot in your leg (Deep Vein Thrombosis)?
·	Blood clot in your lung (Pulmonary Embolism)?
·	None

5.	While still in the hospital after your recent surgery, did you have PROBLEMS WITH YOUR LUNGS & BREATHING:  (Fill in all that apply)
·	You stopped breathing (respiratory arrest)?
·	You were placed on a breathing machine because you were struggling to breathe on your own (respiratory failure)?
·	An infection in your lungs (pneumonia)?
·	None

6.	While still in the hospital after your recent surgery, did you have PROBLEMS WITH YOUR KIDNEYS OR INTESTINE:  (Fill in all that apply)
·	Kidney failure and you needed kidney dialysis?
·	GI bleed (internal bleeding from your stomach or intestine)?
·	Stomach or intestinal ulcer?
·	None

7.	While still in the hospital after your recent surgery, did you suffer from severe pain that required treatment?  
·	yes
·	No (Skip to question 9)

8.	Were you satisfied with the treatment given to you for your severe pain?
·	Yes
·	No
9.	While still in the hospital after your recent surgery, did you have ANY OTHER PROBLEMS:  (Fill in all that apply)
·	A fall, including a slip or trip in which you lost your balance and landed on the floor or ground or lower level. 
·	Delirium (temporary confusion with problems paying attention or thinking clearly)?
·	Stroke (for example, weakness on one side of the body or difficulty speaking)?
·	Nerve injury/paralysis related to your procedure?
·	Infection in the surgical wound?
·	Other (specify):__________________________________________________________
·	None

10.	After leaving the hospital following your surgery, did you receive medical care in any of the following locations? (please check all that apply)
·	Outpatient clinic visit
·	Urgent care center visit
·	Emergency room visit
·	Admitted to a hospital
·	Admitted to a long-term care hospital or inpatient rehabilitation facility
·	Underwent another surgery in an operating room
·	Other location
·	I did not receive any care 

11.	After leaving the hospital following your surgery, did you receive medical care for any of the following reasons? (please check all that apply)
·	Routine medical care RELATED to your surgery
·	Routine medical care NOT RELATED to your surgery
·	Non-routine medical care RELATED TO your surgery
·	Non-routine medical care NOT RELATED to your surgery
·	Radiation therapy or chemotherapy
·	I received care for a different reason
·	I did not receive any care 

12.	After leaving the hospital, did you receive medical treatment FOR PROBLEMS WITH YOUR HEART? (Fill in all that apply)	
·	Heart attack? 										
·	Your heart stopped beating (cardiac arrest)? 					
·	Heart failure (congestive heart failure)? 						
·	Abnormal heart rhythm such as atrial fibrillation? 					
·	Severe pain coming from your heart (angina)?
 	
13.	After leaving the hospital, did you seek medical treatment FOR PROBLEMS WITH BLOOD CLOTS? (Fill in all that apply)		
·	Blood clot in your leg (Deep Vein Thrombosis)? 							
·	Blood clot in your lung (Pulmonary Embolism)? 	
·	None

14.	After leaving the hospital, did you receive medical treatment FOR PROBLEMS WITH YOUR LUNGS OR BREATHING? (Fill in all that apply)		
·	You stopped breathing (respiratory arrest)? 						
·	You were placed on a breathing machine because you were struggling to breathe on your own (respiratory failure)? 									
·	An infection in your lungs (pneumonia)?	
·	None

15.	After leaving the hospital, did you receive medical treatment FOR PROBLEMS WITH YOUR KIDNEYS OR INTESTINE? (Fill in all that apply)
·	Kidney failure and you needed kidney dialysis? 							
·	GI bleed (internal bleeding from your stomach or intestine)? 			
·	Stomach or intestinal ulcer? 	
·	None

16.	After leaving the hospital, did you receive medical treatment for severe pain? 	
·	Yes 	
·	No 

17.	After leaving the hospital, did you receive medical treatment FOR ANY OTHER PROBLEMS? (Fill in all that apply) Need a discussion about free text from PG to see if other items should be added here.
·	Stroke (for example, weakness on one side of the body or difficulty speaking)?
·	Nerve injury/paralysis related to your procedure?
·	Infection in the surgical wound?	
·	Other (specify):__________________________________________
·	None

18.	Since your surgery, how many times have you had a fall, including a slip or trip in which you lost your balance and landed on the floor or ground or lower level?
·	Zero (0) (Please skip to question #20)
·	One time (1)
·	Two times (2)
·	Three or more (>2)

19.	Did your fall result in any of the following? (Circle all that apply)
·	No injury
·	Bruising, sprains or cuts
·	Reduced mobility
·	A fear of falling
·	Severe pain
·	Injury causing you to seek medical treatment
·	Broken bone
·	Head injury
·	A change from independent living to assisted living 

20.	How does your CURRENT use of pain medications compare to your use BEFORE your surgery?
·	I take LESS pain medication than before my procedure
·	I take MORE pain medication than before my procedure
·	I take the SAME amount of pain medication as I did before my procedure
·	I take pain medications now, but did not before my procedure
·	I am not taking pain medications now, and did not before my procedure


21.	Did you have general anesthesia for your surgical procedure? 
·	Yes	
·	No (skip to Question # 23)	
·	I'm not sure

22.	Do you remember anything in between going to sleep and waking up from your anesthesia? 
·	Yes	
·	No (skip to Question # 23)

23.	Was this experience distressing to you?	
·	Yes	
·	No


	


24.	In general, would you say your health is:  
·	Excellent	 
·	Very good		
·	Good	  
·	Fair	  	
·	Poor

25.	Does your health now limit you in moderate activities, such as moving a table, pushing a vacuum cleaner, bowling, or playing golf? If so, how much?	
·	Yes, limited a lot	
·	Yes, limited a little	
·	No, not limited at all

26.	Does your health now limit you in climbing several flights of stairs? If so, how much?	
·	Yes, limited a lot	
·	Yes, limited a little	
·	No, not limited at all

27.	As a result of your physical health, during the past 4 weeks, have you accomplished less than you would like with your work or other regular daily activities? 
·	No, none of the time 
·	Yes, a little of the time
·	Yes, some of the time
·	Yes, most of the time	
·	Yes, all of the time

28.	 As a result of your physical health, during the past 4 weeks, were you limited in the kind of work or other activities? 
·	No, none of the time
·	Yes, a little of the time
·	Yes, some of the time
·	Yes, most of the time	
·	Yes, all of the time
		
29.	 As a result of any emotional problems (such as feeling depressed or anxious), during the past 4 weeks, have you accomplished less than you would like with your work or other regular daily activities? 
·	No, none of the time
·	Yes, a little of the time
·	Yes, some of the time
·	Yes, most of the time	
·	Yes, all of the time

30.	 As a result of any emotional problems (such as feeling depressed or anxious), during the past 4 weeks, have you not done work or other activities as carefully as usual? 	
·	No, none of the time
·	Yes, a little of the time
·	Yes, some of the time
·	Yes, most of the time	
·	Yes, all of the time

31.	During the past 4 weeks, how much did pain interfere with your normal work (including both work outside the home and housework)?  
·	Not at all        
·	A little bit        
·	Moderately        
·	Quite a bit        
·	Extremely

32.	 How much of the time during the past 4 weeks have you felt calm and peaceful? 	
·	All of the time
·	Most of the time	
·	A good bit of the time	
·	Some of the time	
·	A little bit of the time	
·	None of the time

33.	How much of the time during the past 4 weeks did you have a lot of energy?
·	All of the time
·	Most of the time	
·	A good bit of the time	
·	Some of the time	
·	A little bit of the time	
·	None of the time

34.	How much of the time during the past 4 weeks have you felt downhearted and blue?	
·	All of the time
·	Most of the time	
·	A good bit of the time	
·	Some of the time	
·	A little bit of the time	
·	None of the time

35.	How much of the time during the past 4 weeks has your physical health or emotional problems interfered with your social activities (like visiting with friends, relatives, etc.)?
·	All of the time
·	Most of the time	
·	Some of the time	
·	A little bit of the time	
·	None of the time

36.	Compared to one year ago, how would you rate your physical health in general now?
·	Much better
·	Slightly better
·	About the same
·	Slightly worse
·	Much worse

37.	Compared to one year ago, how would you rate your emotional problems now? (Such as feeling anxious, depressed or irritable) 
·	Much better
·	Slightly better
·	About the same
·	Slightly worse
·	Much worse

38.	Currently, do you have any pain in the surgical incision or area related to your surgery? 
·	Yes 
·	No (Skip to question 42 if your answer is no)

39.	Did the pain start after surgery?
·	Yes
·	No

40.	On a scale of zero to ten, with zero being no pain and ten being the worst pain, please fill in your average pain level during the last week.

0	   1	    2	    3	    4	    5	  6	 7         8	9	10          
                                                                               	
	
41.	If you have pain in surgical area, do you any of the following symptoms (check all that apply)
·	Numbness
·	Decrease sensation to cold or touch
·	Increased sensation to cold or touch
		
42.	 In the past 7 days has your thinking has been slow?
·	Never
·	Rarely (Once) 
·	Sometimes (Two or three times)	
·	Often (About once a day)
·	Very often (Several times a day)

43.	In the past 7 days has it seemed like your brain was not working as well as usual?	
·	Never
·	Rarely (Once) 
·	Sometimes (Two or three times)	
·	Often (About once a day)
·	Very often (Several times a day)

44.	 In the past 7 days have you had to work harder than usual to keep track of what you were doing?
·	Never
·	Rarely (Once) 
·	Sometimes (Two or three times)	
·	Often (About once a day)
·	Very often (Several times a day)

45.	 In the past 7 days have you had trouble shifting back and forth between different activities that require thinking?
·	Never
·	Rarely (Once) 
·	Sometimes (Two or three times)	
·	Often (About once a day)
·	Very often (Several times a day)

46.	In the past 7 days has your mind been as sharp as usual?
·	Not at all
·	A little bit
·	Somewhat	
·	Quite a bit
·	Very much

47.	In the past 7 days has your memory been as good as usual?
·	Not at all
·	A little bit
·	Somewhat	
·	Quite a bit
·	Very much

48.	In the past 7 days has your thinking been as fast as usual?
·	Not at all
·	A little bit
·	Somewhat	
·	Quite a bit
·	Very much

49.	In the past 7 days have you been able to keep track of what you are doing, even if you are interrupted?
·	Not at all
·	A little bit
·	Somewhat	
·	Quite a bit
·	Very much


50.	In relation to feeding yourself, you are…
·	unable
·	needing some help (i.e. cutting, spreading butter)
·	independent

51.	In relation to bathing/showering, you are…
·	dependent
·	independent  

52.	In relation to grooming, you are…
·	needing some help with personal care
·	independent (i.e. brushing hair, brushing teeth, shaving) 

53.	In relation to dressing, you are…
·	dependent
·	needing some help, but can do about half unaided
·	independent (including buttons, zips, laces, etc.) 

54.	In relation to your bowels (defecation), you are…
·	incontinent/unable to control bowels (or need to be given enemas)
·	having occasional accidents
·	continent/able to control bowels

55.	In relation to your bladder (urination), you are…
·	incontinent/unable to control bladder (or catheterized and unable to manage alone)
·	having occasional accidents
·	continent/able to control bladder

56.	In relation to using the toilet, you are…
·	dependent
·	needing some help, but can do some things alone
·	independent (on and off the toilet, dressing, wiping) 

57.	In relation to transferring from a bed to a chair and back, you are…
·	unable (no sitting balance)
·	needing major help but are able to sit (one or two people physically helping)
·	needing minor help (verbal encouragement or physical help)
·	independent

58.	In relation to your mobility (walking) on level surfaces, you are…
·	immobile (unable to walk or move about) for less than 50 yards
·	wheelchair independent, including corners, greater than 50 yards
·	walking with the help of one person (either verbal encouragement or physical help) greater than 50 yards
·	independent (with or without a cane or walker) greater than 50 yards 

59.	In relation to climbing a flight of stairs, you are…
·	unable
·	needing help (verbal encouragement, physical help, carrying aid)
·	independent
