# Supplementary material for: Assessing change in patient-reported quality of life after elective surgery: protocol for an observational comparison study
Source: F1000Res. 2016 May 24;5:976. [Version 1] doi: 10.12688/f1000research.8758.1 (PMC5017283; doi:10.12688/f1000research.8758.1)
Supplement: Supplementary file 3 [file f1000research-5-9425-s0002.tgz › 11629030-8ed4-4ec6-b385-dec6cc1106a0.rtf]

	FOR IRB USE ONLY

IRB ID #: 201203088
APPROVAL DATE: 08/31/15
RELEASED DATE: 08/31/15
EXPIRATION DATE: 03/02/16	


INFORMED CONSENT DOCUMENT

Project Title:	SATISFY-SOS: Systematic Assessment and Targeted Improvement of Services Following Yearlong Surgical Outcomes Surveys

Principal Investigator:	Michael Avidan, MD

Research Team Contact:	Sherry McKinnon, Project Manager: 314-286-1768


This consent form describes the research study and helps you decide if you want to participate.  It provides important information about what you will be asked to do during the study, about the risks and benefits of the study, and about your rights as a research participant. 
·	If you have any questions about anything in this form, you should ask the research team for more information.  
·	You may also wish to talk to your family or friends about your participation in this study.
·	Do not agree to participate in this study unless the research team has answered your questions and you decide that you want to be part of this study. 

WHAT IS THE PURPOSE OF THIS STUDY?
The Department of Anesthesiology at Washington University is conducting a project to track the health and well-being of patients after surgery. To do this, we are developing a research database that includes survey feedback from patients in addition to existing medical record information. This database will be used to conduct future research and to improve patient care. You are being asked to participate because you are planning to undergo a procedure requiring anesthesia services at a BJC HealthCare treatment facility.

WHAT WILL HAPPEN DURING THIS STUDY?
You will be asked to complete three surveys. You will complete the first survey today, which asks about your quality of life.  You will complete a survey at approximately 30 days after your surgery and again at approximately 1 year after your surgery. These surveys will ask questions about your quality of life, your specific health, your pain level, and your memory and thinking. You are free to skip any questions that you prefer not to answer. You will be contacted either by email, over telephone by a research assistant, or receive a paper survey in the mail.  

During your hospital stay, you may be visited by a research team member to answer specific questions focusing on your level of alertness and on your pain level.

The research team will access your medical record. Your medical record contains private, protected health information, which is information that can personally identify you such as your name and Social Security Number. The research team uses this information to verify completed surveys and to follow when patients have medical complications after their procedures. We will collect information about today's physical exam, about your surgery and anesthesia and about your recovery.  The research team only collects existing medical information and will not create or add any information to your medical record. 

HOW MANY PEOPLE WILL PARTICIPATE?
Approximately 40,000 people will take part in this study conducted by investigators at Washington University.  

HOW LONG WILL I BE IN THIS STUDY?
If you agree to take part in this study, your direct involvement will last for approximately 1 year from the time of your surgery. If you happen to undergo multiple surgeries during the enrollment period for this project, you will receive a set of surveys for each surgery. The research team may review medical records up to five years for long-term follow-up.

WHAT ARE THE RISKS OF THIS STUDY?
One rare risk of participating in this study is that confidential information about you might be accidentally disclosed. We take privacy protections very seriously and will do our best to keep the information about you secure. We think the risk of accidental disclosure is very low. 

WHAT ARE THE BENEFITS OF THIS STUDY?
We don't know if you will benefit from being in this study.  However, your participation will provide important information on how our patients are doing and feeling after they leave the hospital. The information from your survey and other surveys will allow us to improve the quality of care we deliver to the community served by BJC HealthCare and Washington University. You will not have any costs and you will not be paid for being in this research study.

WHO IS FUNDING THIS STUDY?
The Washington University Department of Anesthesiology and the Barnes-Jewish Hospital Research Foundation are funding this project. 

HOW WILL YOU KEEP MY INFORMATION CONFIDENTIAL?
We will keep your participation in this research study confidential to the extent permitted by law.  However, it is possible that other people such as those indicated below may become aware of your participation in this study and may inspect and copy records pertaining to this research.  Some of these records could contain information that personally identifies you. 
·	Government representatives, (including the Office for Human Research Protections) to complete federal or state responsibilities
·	Hospital or University representatives, to complete Hospital or University responsibilities
·	Press Ganey, processing of mail and phone surveys
·	Solutions Data Systems, processing of baseline quality of life surveys 
·	Washington University's Institutional Review Board (a committee that oversees the conduct of research involving human participants.) The Institutional Review Board has reviewed and approved this study. 
·	We may share de-identified health information such as demographics, medical problems, surgical outcomes and complications and results from the surveys with third parties such as insurance companies and health associations to improve health care. 

To help protect your confidentiality your completed survey will be coded with a unique ID. All electronic data will be hosted on a password-protected, firewall-secured server that is only accessible to the research team through password-protected departmental computers. If we write a report or article about this study or share the study data set with others, we will do so in such a way that you cannot be directly identified.

Protected Health Information (PHI) is health information that identifies you. PHI is protected by federal law under HIPAA (the Health Insurance Portability and Accountability Act). To take part in this research, you must give the research team permission to use and disclose (share) your PHI for the study as explained in this consent form.  The research team will follow state and federal laws and may share your health information with the agencies and people listed under the previous section titled, “How will you keep my information confidential?”.

Once your health information is shared with someone outside of the research team, it may no longer be protected by HIPAA.  

The research team will only use and share your information as talked about in this form. When possible, the research team will make sure information cannot be linked to you (de-identified). Once information is de-identified, it may be used and shared for other purposes not discussed in this consent form. If you have questions or concerns about your privacy and the use of your PHI, please contact the University's Privacy Officer at 866-747-4975. 

Although you will not be allowed to see the study information, you may be given access to your health care records by contacting your health care provider. 

If you decide not to sign this form, it will not affect
·	your treatment or the care given by your health provider.
·	your insurance payment or enrollment in any health plans.  
·	any benefits to which you are entitled.
However, it will not be possible for you to take part in the study.

If you sign this form:
·	You authorize the use of your PHI for this research
·	Your signature and this form will not expire as long as you wish to participate.
·	You may later change your mind and not let the research team use or share your information (you may revoke your authorization). 
·	To revoke your authorization, complete the withdrawal letter, found in the Participant section of the Human Research Protection Office website at http://hrpo.wustl.edu/participants/withdrawing-from-a-study/ or you may request that the Investigator send you a copy of the letter.   			
o	If you revoke your authorization:
§	The research team may only use and share information already collected for the study.
§	Your information may still be used and shared if necessary for safety reasons. 
§	You will not be allowed to continue to participate in the study.

Can we contact you by email?
We would like to contact you by email so you can complete the survey online through a secure link.  Only the research team will have access to your email communications.  We will only communicate by email to send you the secure survey link.  If you have any questions or need to contact us, please contact the Project Manager at the number listed on the front page of this consent form.

The survey questions ask about your health and well-being but do not contain health information that can directly identify you. Your answers to the questions will also not directly identify you.

IS BEING IN THIS STUDY VOLUNTARY?
Taking part in this research study is completely voluntary.  You may choose not to take part at all.  If you decide to be in this study, you may stop participating at any time.  If you decide not to be in this study, or if you stop participating at any time, you won't be penalized or lose any benefits for which you otherwise qualify. You may withdraw by telling the study team you are no longer interested in participating in the study or you may send in a withdrawal letter. A sample withdrawal letter can be found at http://hrpo.wustl.edu/participants under Withdrawing from a Research Study.

WHAT IF I HAVE QUESTIONS?
We encourage you to ask questions. If you have any questions about the research study itself, please contact: Sherry McKinnon, Project Manager at 314-286-1768. If you have questions, concerns, or complaints about your rights as a research participant, please contact the Human Research Protection Office, 660 South Euclid Avenue, Campus Box 8089, St. Louis, MO  63110, 1-(800)-438-0445 or email hrpo@wustl.edu. General information about being a research participant can be found by clicking “Participants” on the Human Research Protection Office web site, http://hrpo.wustl.edu.  To offer input about your experiences as a research subject or to speak to someone other than the research staff, call the Human Research Protection Office at the number above.


This consent form is not a contract. It is a written explanation of what will happen during the study if you decide to participate. You are not waiving any legal rights by agreeing to participate in this study. 

Your signature indicates that this research study has been explained to you, that your questions have been answered, and that you agree to take part in this study.  You will receive a signed and dated copy of this form.
Do not sign this form if today's date is after EXPIRATION DATE: 03/02/16.


________________________________________________________________________
(Signature of Participant)					(Date)


___________________________________________
(Participant's name – printed)

Statement of Person Who Obtained Consent
The information in this document has been discussed with the participant or, where appropriate, with the participant's legally authorized representative.  The participant has indicated that he or she understands the risks, benefits, and procedures involved with participation in this research study.


________________________________________________________________________
(Signature of Person who Obtained Consent)			(Date)


___________________________________________
(Name of Person who Obtained Consent - printed)


					Patient sticker
Patient email address 
FIRST PART of the e-mail address (before the @ sign)
Print in ALL CAPS and darken punctuation marks.  Example:	J
O
H
N
_
D
O
E
9
9

	

																						

SECOND PART of the e-mail address (after the @ sign).  CHECK ONE:
  @AOL.COM
  @ATT.NET
  @CHARTER.NET
  @COMCAST.NET	  @GMAIL.COM
  @HOTMAIL.COM
  @MSN.COM
  @SBCGLOBAL.NET	  @WUSTL.EDU
  @YAHOO.COM
  @YMAIL.COM	
  OTHER:  Print in ALL CAPS and darken punctuation marks.  
@																					
	
