# Supplementary material for: Assessing change in patient-reported quality of life after elective surgery: protocol for an observational comparison study
Source: F1000Res. 2016 May 24;5:976. [Version 1] doi: 10.12688/f1000research.8758.1 (PMC5017283; doi:10.12688/f1000research.8758.1)
Supplement: Peer review history

# Peer review: Referee response for version 1

**DOI:** 10.5256/f1000research.9425.r15800

**Published:** 2016-09-08

**Authors:** Meghan Lane-Fall

**Affiliations:** 1 Department of Anesthesiology and Critical Care, University of Pennsylvania Perelman School of Medicine, Philadelphia, PA, USA

In this report, Kronzer and colleagues detail a study protocol for an observational study of patient quality of life after elective surgery. They intend to use two methods to assess quality of life: (1) a validated scale and (2) a simple closed-ended question about whether their quality of life is better, worse, or the same as that before surgery. In the interest of research transparency, I applaud the authors on publishing their study protocol in advance of proceeding with their work. This will allow reviewers and readers of subsequent reports to determine whether the investigators adhered to their study protocol.

**Manuscript comments:**

 The manuscript is clearly written and easy to understand. The data collection approach and analytic procedures are reasonable, and the authors have adequately addressed potential threats to validity (e.g. non-response bias). It is unfortunate that limited English speakers are excluded from the study population, but this is a limitation of the registry overall, not just this sub-study.

 Major criticisms:

- The authors state in their Limitations section that some of the surveys are completed as late as 120 days after surgery (instead of 30 days after). I worry that the relationship between the VR-12 score and the single global QOL question may not be consistent across time, which could bias the results. Have the authors considered restricting their sample to patients who complete the follow-up survey within a certain range of time (e.g. 20-40 days)?

Minor criticisms:

- I would define VR-12 earlier in the manuscript. It is currently in the Data section, but should be moved up to its first mention in the Methods section.
- In the Data section, the authors state that the informaticist "performs rigorous data validation on each queried variable", but do not specify what the data validation procedures are. Some limited additional detail here would be useful.

**Instrument comments:**

 Minor criticism:

- In the baseline instrument, both "circle all" (Question 1) and "check all" (Question 2) are used - these should be the same for consistency.

**References:**

- In Reference 19, the title is incorrect. "Methodological" should be "Methodologic", and the citation should be updated with publication information: Anesthesiology. 2016 Sep;125(3):495-504. doi: 10.1097/ALN.0000000000001217.

I have read this submission. I believe that I have an appropriate level of expertise to confirm that it is of an acceptable scientific standard.

# Peer review: Referee response for version 1

**DOI:** 10.5256/f1000research.9425.r15801

**Published:** 2016-09-05

**Authors:** Anna Woodbury

**Affiliations:** 1 Department of Anesthesiology and Pain Management, Veteran Affairs Medical Center, Atlanta, GA, USA

The primary objective of this study is to perform a sub-study of the Systematic Assessment and Targeted Improvement of Services Following Yearlong Surgical Outcomes Surveys (SATISFY-SOS) study by comparing a validated quality of life measure (VR-12) to a self-reported "global" quality of life question.   Secondary aims include comparing mental and physical quality of life pre- and post- surgery (Aim 2) and to describe these based on types of surgeries (Aim 3).  The authors feel this is important because self-reported measures are subject to recall bias, and yet are more commonly used.

 Major criticisms:  None

 Minor criticisms:

1. Co-morbidities (such as depression, Alzheimer's, or other psychiatric or neurologic illness) and their treatment or lack of treatment may affect quality of life scores.  Will these be accounted for and will the patient be monitored?  For example, if a patient suffers from depression, undergoes surgery, and is started on an antidepressant after surgery, this would potentially affect quality of life, but is not directly related to the surgery itself.  I would like to know how this type of confounding variable will be addressed.
2. The validated tool used should be compared also with the PROMIS measures, which are essentially individually validated questions, often reliant on patient self-report. Would consider adding these.

I have read this submission. I believe that I have an appropriate level of expertise to confirm that it is of an acceptable scientific standard.
